# CRISPR/Cas9-Mediated Insertion of loxP Sites in the Mouse *Dock7* Gene Provides an Effective Alternative to Use of Targeted Embryonic Stem Cells

**DOI:** 10.1534/g3.116.030601

**Published:** 2016-05-11

**Authors:** Kathleen A. Bishop, Anne Harrington, Evguenia Kouranova, Edward J. Weinstein, Clifford J. Rosen, Xiaoxia Cui, Lucy Liaw

**Affiliations:** * Center for Molecular Medicine, Maine Medical Center Research Institute, Scarborough, Maine 04074; † Horizon Discovery, St. Louis, Missouri 63146

**Keywords:** *Dock7*, CRISPR/Cas9, KOMP ES cells

## Abstract

Targeted gene mutation in the mouse is a primary strategy to understand gene function and relation to phenotype. The Knockout Mouse Project (KOMP) had an initial goal to develop a public resource of mouse embryonic stem (ES) cell clones that carry null mutations in all genes. Indeed, many useful novel mouse models have been generated from publically accessible targeted mouse ES cell lines. However, there are limitations, including incorrect targeting or cassette structure, and difficulties with germline transmission of the allele from chimeric mice. In our experience, using a small sample of targeted ES cell clones, we were successful ∼50% of the time in generating germline transmission of a correctly targeted allele. With the advent of CRISPR/Cas9 as a mouse genome modification tool, we assessed the efficiency of creating a conditional targeted allele in one gene, *dedicator of cytokinesis 7* (*Dock7*), for which we were unsuccessful in generating a null allele using a KOMP targeted ES cell clone. The strategy was to insert loxP sites to flank either exons 3 and 4, or exons 3 through 7. By coinjecting Cas9 mRNA, validated sgRNAs, and oligonucleotide donors into fertilized eggs from C57BL/6J mice, we obtained a variety of alleles, including mice homozygous for the null alleles mediated by nonhomologous end joining, alleles with one of the two desired loxP sites, and correctly targeted alleles with both loxP sites. We also found frequent mutations in the inserted loxP sequence, which is partly attributable to the heterogeneity in the original oligonucleotide preparation.

The International Knockout Mouse Consortium (IKMC), and participating organizations, are using both targeted deletion and knockout-first allele strategies to generate embryonic stem (ES) cell lines with both knockout and conditional knockout potential (Friedel *et al.* 2007; Lloyd 2011). To date, investigators have had success with germline transmission of the targeted clones, providing valuable tools to the research community (Coleman *et al.* 2015; Cotton *et al.* 2015). Since initial offerings of targeted ES cell clones, the KOMP project has moved forward to the production, gene expression analysis (West *et al.* 2015), and phenotyping of thousands of mouse strains (Ring *et al.* 2015), and the corresponding database management system to allow data access for researchers (Ringwald *et al.* 2011; Koscielny *et al.* 2014). Although the majority of the mutant alleles that have generated mouse strains are correctly targeted alleles, thorough molecular characterization has identified limitations that users need to be of aware of in characterizing their own particular lines of interest. Mouse strains generated from the EUCOMM/KOMP-CSD collection have previously been subjected to a rigorous quality control screen to confirm correct cassette targeting and potential for conditional deletion. Analyzing 731 mouse strains from this resource, 86% of resultant mice had the correct targeting and cassette structure, and 97% of these retained sequences required for conditional inactivation of the allele (Ryder *et al.* 2013). The unexpected events that precluded generation of a successful allele included incorrect targeting, deletions in the 5′ end of the targeting cassette, variability of success depending on the ES cell clone initially used for targeting, and evidence for mixed populations of ES cells in the targeted “clone” (Ryder *et al.* 2013). As resource users, it is also important to consider that there are additional limitations that may arise by problematic chimera generation and/or germline transmission (Pacholczyk *et al.* 2008; Coleman *et al.* 2015). While the study of Ryder *et al.* (2013) characterized alleles that had been transmitted successfully, aggregate germline transmission rates appear to be around 50% [KOMP repository germline transmission rates and reference (Cotton *et al.* 2015)].

With its practical application and successes with modification of the mouse genome, CRISPR/Cas9 technology is an alternative for efficient conditional gene targeting (Wang *et al.* 2013; Yang *et al.* 2013; Cong *et al.* 2013). The Jaenisch lab demonstrated a 20% targeting efficiency of introducing one loxP site into two separate genes (Tet1 and Tet2), and ∼16% efficiency in targeting two loxP sites into one allele of the *Mecp2* gene using two targeting small guide RNAs (sgRNA), and two loxP-containing oligonucleotides (Yang *et al.* 2013). An alternate strategy was used to introduce two loxP sites into the *Ispd* locus using a single DNA template containing the floxed exon with flanking homologous arms (1.9 kb each) using paired Cas9 nickase and two sgRNAs (Lee and Lloyd 2014), although the efficiency was relatively low (8% of live pups born). To generate a conditional *dedicator of cytokinesis 7* (*Dock7*) allele, we targeted sgRNAs to insert loxP sites to flank either exons 3 and 4, or exons 3 and 7. Using this strategy, we generated a variety of alleles, including a correctly targeted, novel, conditional null allele of the *Dock7*gene.

## Materials and Methods

### Reagents

General chemicals and reagents were purchased from Sigma-Aldrich (St. Louis, MO) and ThermoFisher Scientific (Waltham, MA). HEPES-buffered DMEM (12430) and TOPO TA cloning kit (K461020) were purchased from ThermoFisher Scientific (Waltham, MA). Giemsa stain (GS500), Jumpstart Taq ReadyMix (P2893), and mineral oil (M8410) were obtained from Sigma-Aldrich (St. Louis, MO). Fetal bovine serum (35-074-CV), and trypsin (25-052) were purchased from Corning Life Sciences (Corning, NY). The hiScribeTM T7 quick high yield RNA synthesis kit (E2050S), Phusion high fidelity polymerase (M0530S), and proteinase K (P8102) were obtained from New England Biolabs (Ipswich, MA). QIAquick PCR purification kits (28104), QIAquick gel extraction kits (28704), and QIAprep spin miniprep kits (27106) were purchased from Qiagen (Germantown, MD). EmbryoMax M2 medium (MR015P5D), ESGRO leukemia inhibitory factor (ESG1107), and colcemid (234109) were obtained from EMD Millipore (Billerica, MA). Chorionic gonadotropin and pregnant mare serum were purchased from the National Hormone and Peptide Program (NHPP), Harbor-UCLA Medical Center (Torrance, CA). Penicillin/streptomycin (SV30010) from Hyclone (Logan, UT), MessageMAX T7 ARCA-capped message transcription kit (C-MMA60710) from Cellscript (Madison, WI), EMEM media (ATCC-30-2003) from ATCC (Manassas, VA), SF Cell Line 96-well Nucleofector Kit (V4SC-2096) from Lonza (Basel, Switzerland), and the QuickExtract DNA extraction solution (QE09050) from Epicentre (Madison, WI) were obtained. The Surveyor mutation assay kit (S100) and ultramer oligonucleotides were purchased from Integrated DNA Technologies (Coralville, IA). AccuStart II PCR SuperMix (95137) was purchased from Quanta Biosciences (Gaithersburg, MD), MasterTaq (2200230) was obtained from 5 PRIME (Gaithersburg, MD), and Terra PCR direct polymerase mix (639270) was purchased from Clontech (Mountain View, CA).

### Mouse strains

C57BL/6J and B6.Cg-Tg(Sox2-cre)1Amc/J (Sox2-Cre, stock 8454) mice were purchased from the Jackson Laboratory (Bar Harbor, ME); C57BL/6NCrl (C57BL/6N) mice were purchased from Charles River (Wilmington, MA), and Swiss Webster mice were purchased from Taconic (Taconic, Hudson, NY). Mice were housed in the barrier, AAALAC-accredited animal facility at Maine Medical Center Research Institute. All studies were reviewed and approved by the Institutional Animal Care and Use Committee of Maine Medical Center, and followed National Institutes of Health (NIH) guidelines for the Care and Use of Laboratory Animals.

### Culture of ES cell clones

Targeted ES cell lines available via KOMP were purchased and cultured according to the protocol provided (Pettitt *et al.* 2009; https://www.komp.org/protocols.php). Briefly, ES cells were cultured in HEPES-buffered DMEM, 5% FBS, and 1% penicillin/streptomycin, and grown on a feeder layer of mitotically inactivated mouse embryonic fibroblasts with leukemia inhibitory factor at 37°, 5% CO_2_. For subculture, ES cell colonies were treated with 0.25% trypsin into a single cell suspension and replated onto a fresh plate with a mouse embryonic fibroblast feeder layer.

### Clonal expansion of ES cell lines and chromosome counts

Individual ES cell colonies were picked, trypsinized, and propagated for DNA collection and cryopreservation. Chromosome counts were performed by treating exponentially growing cultures with 0.02 mg/ml colcemid in fresh growth medium for 1 hr prior to trypsinization. Cells were pelleted and treated with 0.56% KCl for 6 min, then pelleted, and fixed with 3:1 methanol:acetic acid. After two additional washes with fixative, cells were dropped onto glass slides and stained with Giemsa. Chromosomes were visualized at 1000 × magnification with light microscopy, photographed, and total chromosomes were counted for a minimum of 10 spreads per slide.

### Microinjection of ES cells

Three-wk-old C57BL/6N female mice were injected with 5 IU/mouse of pregnant mare serum followed 48 hr later with 5 IU/mouse of human chorionic gonadotropin, and mated to C57BL/6N males. At 3.5 d post coitum (dpc), the females were killed and the blastocysts were flushed from the uterine horn with microinjection medium (5% FBS added to HEPES-buffered D-MEM). Blastocysts were kept warm at 37° until injection, when both blastocysts and a single cell suspension of ES cells were placed in 50 μl droplets of microinjection medium covered in mineral oil for injection, similar to the procedure described in Behringer *et al.* (2014). The blastocysts were injected using Narishige oil hydraulic micromanipulators using VacuTip holding capillaries (930-00-101-5), and beveled TransferTips (930-00-104-0) purchased from Eppendorf (Hamburg, Germany). ES cells were aspirated into a TransferTip backfilled with microinjection medium. Each blastocyst received 10–12 ES cells. After injection, blastocysts were incubated at 37°, 5% CO_2_ in injection medium until implantation the same afternoon. Finally, 12–16 injected blastocysts were divided equally between two uterine horns of a 2.5 dpc pseudopregnant Swiss Webster female. Injections of morulae followed a similar protocol. Briefly, morulae were collected at 2.5 dpc, injected with 10–12 ES cells, and 12–16 morulae were divided equally between two uterine horns of a 0.5 dpc Swiss Webster female. For ES cell lines derived from the JM8A3 parental line, chimerism was detected based on agouti coat color.

### CRISPR/Cas9 reagents

Cas9 mRNA was transcribed *in vitro* using a MessageMax T7 kit and linearized plasmid carrying a T7 promoter and a human codon-optimized Cas9 open reading frame as a template (Mali *et al.* 2013). The templates used for *in vitro* transcription of small guide RNAs (sgRNAs) were assembled by PCR, amplifying two overlapping DNA oligonucleotides, containing a T7 promoter, 20-bp spacer sequence, and common backbone primer. The PCR product was then purified using the QIAQuick PCR purification kit and used as a template to *in vitro* transcribe sgRNAs with a HiScribe T7 quick high yield RNA synthesis kit. Following *in vitro* transcription, RNA was purified by ethanol precipitation with 1/10 volume of 3 M sodium acetate. Sequences of sgRNA recognition sites and oligonucleotides used for loxP insertion are listed in Supplemental Material, Table S3. Cas9 mRNA, sgRNAs, and oligonucleotides were mixed immediately prior to injection in TE buffer, and centrifuged for 10 min at 14,000 rpm.

### Validation of CRISPR/Cas9 sgRNAs

sgRNAs were validated in mouse Neuro2a cells stably expressing Cas9 (Kouranova *et al.* 2016). The cells were maintained in EMEM medium supplemented with 10% FBS and 1% penicillin/streptomycin at 37° with 5% CO_2_. All cell transfections were performed with a Nucleofector (Lonza) according to the manufacturer’s 96-well shuttle protocol for respective cell lines. After trypsinization, cells were counted, pelleted and washed twice in Hanks balanced salt solution to minimize nuclease carryover from the growth medium. Specifically, for the mouse Neuro-2a cell line, the solution SF solution (SF Cell Line 96-well Nucleofector Kit) and program 96-DS-137 were used to transfect 2–4 μg sgRNA into 200,000 cells per reaction; 1 μg of a GFP plasmid was transfected for each condition as a control. Transfected cells were collected at various time points post transfection, added into 80 µl of QuickExtract DNA extraction solution, and incubated at 65° for 15 min, and 98° for 3 min to release nucleic acids. Target regions were PCR-amplified using the primers listed for genotyping at insertion sites of loxP4, loxP5, and loxP6 (Table S4), and analyzed using the SURVEYOR mutation assay, in which CRISPR cleavage-mediated modifications via nonhomologous end joining (NHEJ) at a target site result in cleavage of the PCR amplicon of the target region into fragments of predicted sizes. The mixtures were resolved on a 10% polyacrylamide TBE gel Validation of the sgRNAs used in this study are shown in Figure S1.

### SURVEYOR mutation assay

Two Taq polymerase mixes and respective conditions were used interchangeably: AccuStart II PCR SuperMix and JumpStart. The corresponding programs are listed in Table S2; 10 μl of the above PCR reactions were incubated under the following program: 95°, 10 min, 95°–85°, at –2°/sec, 85°–25° at –0.1°/sec. According to the protocol for the SURVEYOR mutation detect kit, 1 μl each of nuclease S (Cel-I) and enhancer were added to digest the above reaction at 42° for 20 min.

### Microinjection of CRISPR/Cas9 reagents

Three-wk-old C57BL/6J female mice were injected with 5 IU/mouse of pregnant mare serum, followed 48 hr later with 5 IU/mouse of human chorionic gonadotropin. The females were then mated to C57BL/6J males. Fertilized oocytes were collected at 0.5 dpc in microinjection medium (5% FBS added to HEPES-buffered D-MEM). Fertilized oocytes were place on a depression slide in 30 µl EmbryoMax M2 medium covered in mineral oil, similar to what was previously described (Behringer *et al.* 2014). Using the Eppendorf Transjector 5246, with in-house pulled glass capillaries, fertilized oocytes were injected into either the pronuclei or cytoplasm with the prepared CRISPR/Cas9 reagents using air-regulated compensation and an injection pressure of 90–115 psi in order to create a continuous flow of reagents (Behringer *et al.* 2014). Due to the continuous flow of reagents, fertilized oocytes with injections into the pronuclei received CRISPR/Cas9 reagents in both pronuclear and cytoplasmic regions. Injected zygotes were incubated at 37°, 5% CO_2_ until transplantation. Approximately nine zygotes were then transferred into each oviduct of the pseudopregnant Swiss Webster females on the afternoon of the injection.

### DNA isolation

For ES cells, DNA was isolated similarly to what has been previously described (Zangala 2007). Briefly, cells were centrifuged and resuspended in 300 µl DNA isolation buffer containing 100 mM NaCl, 50 mM Tris, pH 7.5, 10 mM EDTA, 0.5% SDS, and 0.5 mg/ml proteinase K at 56° for 3 hr. DNA was precipitated with an equal volume of isopropanol, and pelleted by centrifugation. The DNA pellet was washed with 750 µl of 70% EtOH, air dried, and resuspended in water. Mouse toe or tail clips from pups derived from injected embryos were incubated in DNA isolation buffer at 56° overnight, precipitated with an equal volume of isopropanol, and pelleted by centrifugation. The DNA pellet was washed with 750 µl of 70% EtOH, air dried, and resuspended in water. For N1, and additional generations, genomic DNA was isolated by incubation of the tail or toe clip in 300 µl of 50 mM NaOH at 95° for 2 hr followed by neutralization with 30 µl Tris-HCl, pH 8.0 (Truett *et al.* 2000).

### Genotyping

Genomic DNA isolated from either ES cells or mice generated from ES cell injections was amplified using MasterTaq using the primer pairs are listed in Table S1 according to the cycling conditions listed in Table S2. DNA isolated from mice generated from the injection of CRISPR/Cas9 reagents was amplified using either JumpStart Taq ReadyMix or Terra Taq, using the primer pairs listed in Table S4 according to the cycling conditions described in Table S2. Both Taq polymerase mixes were found to produce equivalent results. All PCR reactions were run on a 3% agarose gel to visualize the products.

### DNA sequencing of cloned loxP sites in mice generated from CRISPR/Cas9 injection

DNA isolated from tail/toe clips was amplified using Phusion high fidelity polymerase using primers listed in Table S4 according to the cycling condition described in Table S2. To add TA overhangs, 1.25 U of Terra Taq was added, and samples were incubated at 72° for 10 min. Amplified DNA was then cloned using the TOPO TA cloning kit according to the manufacturer’s protocol. Clones were screened for insertions according to the previously described genotyping protocols, purified using QIAquick PCR purification kits, and the PCR products were sequenced at either the Maine Medical Center Research Institute or the Dana-Farber/Harvard Cancer Center DNA Resource Core.

### DNA sequencing of cloned oligonucleotides

Oligonucleotides were amplified using Phusion high fidelity DNA polymerase with primers Oligo4 F/R and Oligo5 F/R as listed in Table S4, and cycling conditions listed in Table S2, then cloned using the TOPO TA cloning kit according to the kit’s protocol. Clones were screened for insertions and sequenced using the T7 forward primer (Table S4) at the Dana-Farber/Harvard Cancer Center DNA Resource Core.

### Analysis of off-target Cas9 activity

The top 10 potential off-target sites for each sgRNA in the *Dock7* cKO1 and cKO2 models were identified using the Zhang lab algorithm (http://crispr.mit.edu/). Flanking PCR primers designed to amplify 300–600 bp fragments are listed in Table S5, Table S6, and Table S7, with the sequence and genomic coordinates of each potential off-target region. DNA was extracted from N0 mice and C57BL/6J mice by proteinase K digestion, and amplified using JumpStart Taq ReadyMix. Amplified DNA samples were assayed using the SURVEYOR mutation assay according to the protocol listed above. The mixture was resolved on 3% agarose gel for the predicted cutting pattern.

### Data availability

Strains are available upon request. The authors state that all data necessary for confirming the conclusions presented in the article are represented fully within the article.

## Results

### Targeted embryonic stem cell approach

We obtained several targeted ES cell clones generated from multiple members within the International Knockout Mouse Consortium (Table 1) (Skarnes *et al.* 2011). These ES cells were grown and expanded according to the instructions for each particular line, and prepared for injection into C57BL/6N morulae or blastocysts. Host C57BL/6 strains were chosen for the ability to distinguish chimerism based on coat color. Table 1 shows the clones injected, number and sex of chimeras obtained, and success in germline transmission of each allele. Three out of the six lines tested passed through the germline, for a germline transmission rate of 50%. Our results from this small collection of ES cell lines are consistent with other reports using aggregate data (Cotton *et al.* 2015) (KOMP germline transmission data, https://www.komp.org/gltrates2.php).

**Table 1:** Chimera generation and germline transmission from targeted embryonic stem cell clones

Gene	Allele	Clone	Source	Parental ES Cell Line	Embryos Injected	Chimeras Generated	Germline Transmission (# Screened)
*Cav3*	*CAV3^tm1(Komp)Vlcg^*	11604B-F10	KOMP	VGB6	32m, 29b	7	6
*Il17rd*	*Il17rd^tm1a(EUCOMM)Hmgu^*	HEPD0636_8_G08	EUCOMM	JM8A3.N1	40m, 14b	2M,3F	2
*Mir199b*	*Mir199b^tm1(mirKO)Wtsi^*	mirKO_ES_PuDtk_11D9	Sanger miR knockouts	JM8A3	69m, 61b	4M*^a^*	0 (>49)*^a^*
		mirKO_ES_PuDtk_11D12	Sanger miR knockouts	JM8A3	66m		
*Lgr4*	*Lgr4^tm1a(EUCOMM)Hmgu^*	HEPD0729_3_A08	EUCOMM	JM8A3.N1	40m, 32b	4M	0 (no pups born)
*Col1a1*	Commercially developed		Applied StemCell	JM8A3.N1	73m, 25b	3M	1
*Dock7*	*Dock7^tm1a(EUCOMM)Wtsi^*	EPD0821_4_G02	EUCOMM	JM8A3.N1	76m, 56b	6M	0 correctly targeted*^b^*

The source of targeted ES cell lines for the genes shown is indicated. Either morula (m) or blastocysts (b) were injected, and resultant chimeras were bred to monitor germline transmission. For those lines without germline transmission, the total number of pups screened is shown in parentheses. m, morula; b, blastocyst; M, male; F, female.

a Both clones were injected, resulting in only one chimera.

b Please see subsequent results for details on the *Dock7* ^tm1a^ allele.

**Figure 1:**
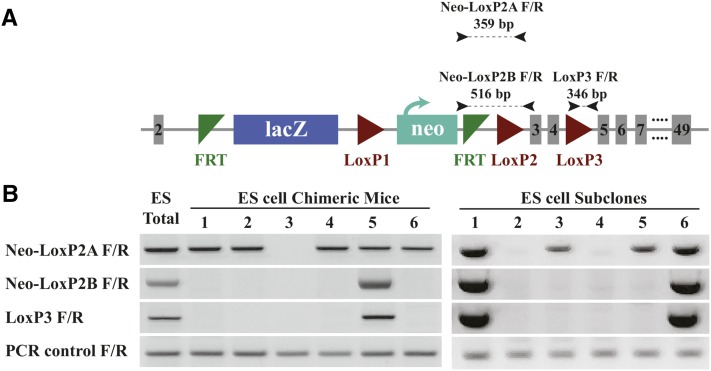
Analysis of the *Dock7* first knockout allele (*Dock7^tm1a^*). (A) Targeting cassette and genotyping primers for the L1L2_Bact_P cassette inserted at the *Dock7* gene locus. Primers used for genotyping are indicated, with the expected product size. Figure was adapted from IMPC.org (Ringwald *et al.* 2011). (B) Left: the original *Dock7^tm1a^* ES targeted line was used for genomic DNA isolation to evaluate the *Dock7* locus (ES total). Genomic DNA was collected from six chimeric mice generated by injection of the ES cell line, and amplified using the three primer pairs. The *Dock7^tm1a^* ES cells, chimeras generated from ES injection, and ES cell subclones were genotyped at the neomycin (neo)-loxP2 (neo-loxP2A F/R, neo-loxP2B F/R) and loxP3 (loxP3 F/R) regions. PCR control is located in exon 18, ∼68 kb downstream of the cassette insertion. Right: a total of 83 subclones was picked from the parental *Dock7^tm1a^* ES cell population, and DNA isolated from the ES cell subclones was analyzed by PCR. A representative example of six subclones is shown, which reflects the amplification patterns found within the subclones.

For the unsuccessful clones, the desired mouse lines were not achieved for a variety of reasons, including lack of germline transmission (Mir199b), and sterility of chimeras (Lgr4). Of particular interest was the *Dock7^tm1a(EUCOMM)Wtsi^* (*Dock7^tm1a^*) knockout-first allele, which we explored in more detail. The *Dock7^tm1a^* chimeric mice were identified by agouti coat color, and genotyped with primers to the neomycin (neo)-loxP2 region (neo-loxP2A F/R, neo-loxP2B F/R) and the loxP3 region (loxP3 F/R) indicated in Figure 1A. The *Dock7^tm1a^*-targeted ES cell line was used as a positive control for DNA amplification where amplified DNA product was detected for each primer pair tested (Figure 1B, ES total). Out of six chimeras generated, only one was identified with amplification from all three primer pairs, suggesting it contained cells with integration of an intact neo-loxP2 boundary (neo-loxP2A F/R, neo-loxP2B F/R) and loxp3 (loxP3 F/R), similar to the ES cell population generated from the EUCOMM stock (Figure 1B). The other chimeric mice had evidence of cells containing sequences amplified by primer pair neo-loxP2A F/R, but not the other two target sites. This suggested that there could be a deletion of DNA in the cassette, and that there was heterogeneity in our starting ES cell population.

The *Dock7^tm1a^* chimeras were bred to C57BL/6J mice, and the offspring were assessed similarly for coat color and genotype to determine germline transmission of the mutant allele (Table 2). Of the four chimeras that produced offspring, only chimeras 1 and 5 showed evidence of germline transmission by agouti coat color. Chimera 1 showed evidence of germline transmission of only a partial *Dock7^tm1a^* cassette, while chimera 5 produced four agouti offspring, genotyping showed no evidence of the targeting cassette in any of the pups.

**Table 2:** Characteristics of N1 offspring from *Dock7^tm1a^* chimeras

Chimera (N0)	Chimera Sex	Total Offspring (N1)	Agouti Offspring	Offspring with Partial Cassette	Offspring with Intact Cassette
1	Male	47	>10	16	0
2	Male	16	0	0	0
3	Male	0	0	0	0
4	Male	0	0	0	0
5	Male	69	4	0	0
6	Male	8	0	0	0

Chimeric mice generated from the *Dock7^tm1a^* ES cells were identified by their agouti coat color, and bred to C57BL/6J mice. The number of offspring (N1) from each of the chimeric mice is shown, along with numbers of mice with agouti coat color and amplification of the targeting cassette. Mice were scored as having an intact cassette if a product was obtained from each of the three primer pairs: neo-loxP2A F/R, neo-loxP2B F/R, and loxP3 F/R. Mice were scored as having a partial cassette if a product was obtained only from neo-loxP2A F/R, but not neo-loxP2B F/R or loxP3 F/R. An example of genotyping results with the chimeras and ES cell subclones can be observed in Figure 1.

To address the possibility of a heterogeneous population of ES cells in the *Dock7^tm1a^* line, we established 83 clonal subpopulations by individual colony picking from the parental *Dock7^tm1a^* line. These subclones were expanded, and neo-loxP2 and loxP3 regions were screened with the aforementioned primer pairs. The amplification patterns matched what was found in the chimeric mice: some clones had all three products (Figure 1B, subclones 1 and 6), and some had a product only from primer pair neo-loxP2A F/R (subclones 3, 5). We also obtained subclones that yielded no evidence of amplification from any of the three primer pairs (subclones 2 and 4). Collectively, we found evidence for an intact targeting cassette in 38% of the subclones (32/83), and a partial targeting cassette in 55% (46/83) of the subclones. Interestingly, 6% (5/83) were negative using all three primer pairs. The presence of ES cells with multiple genotypes within the *Dock7^tm1a^* subclones confirms the heterogeneous nature of the *Dock7^tm1a^* ES cell population, and explains the generation of chimeras with multiple genotypes.

Two *Dock7^tm1a^* ES cell subclones containing the intact targeting cassette (subclones 1 and 6, Figure 1B), originally isolated from the original 83 subclones, were analyzed for chromosome number. We found that over 50% of cells contained an abnormal number of chromosomes, which is known to significantly decrease the potential for germline transmission (Longo *et al.* 1997). Consistent with our observations of abnormal total chromosomal numbers in this particular clone derived from the JM8A3.N1 ES cell line, other investigators have reported that, of the 151 clones (19.8%) tested from the EUCOMM repository, 30 also contained over 50% of cells with abnormal chromosome numbers (Cotton *et al.* 2015). Furthermore, the same study reported that injection of ES cells lines from the eight clones with over 50% of cells with abnormal chromosome counts did not result in germline transmission of the lines test (Cotton *et al.* 2015). These collective data suggest that the chance of germline transmission of a correctly targeted *Dock7^tm1a^* clone was low. Therefore, we did not pursue the *Dock7^tm1a^* clone or subclones further. Additional clones targeting *Dock7* with conditional knockout potential were not available through the KOMP project at the time these experiments were performed.

### CRISPR/Cas9 for conditional gene targeting of the Dock7 locus

**Figure 2:**
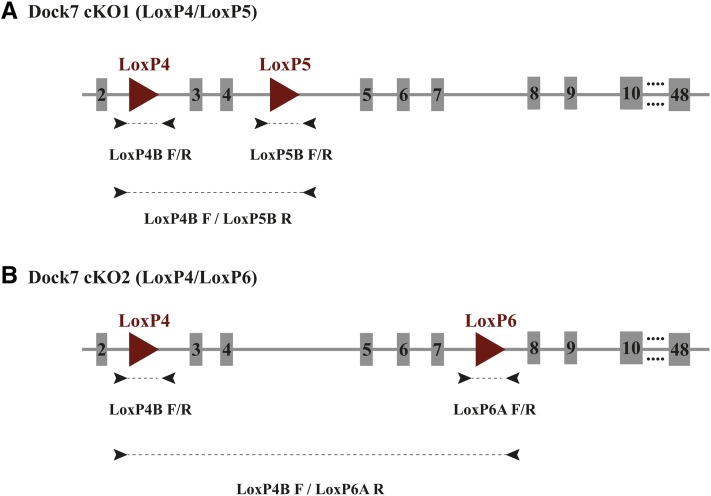
Strategy to generate conditional alleles of *Dock7* by CRISPR/Cas9-mediated targeting. (A) One sgRNA was validated within intron 2 and intron 4, a distance of 2.1 kb apart. Two corresponding oligonucleotides were synthesized with 79–80 bp homology arms flanking the loxP sequences. With the translational start site in exon 1, Cre-mediated recombination of this allele is predicted to result in a premature stop codon due to deletion of exons 3–4 and surrounding intronic sequences. (B) The same upstream sgRNA in intron 2 was also used in combination with an sgRNA targeting in intron 7, with a distance of 5.3 kb in between. The downstream oligonucleotide was designed with a 75–81 bp homology arm flanking loxP and the *Bam*HI restriction site. Cre-mediated recombination of this allele is also expected to generate a premature stop codon upon deletion of exons 3–7.

**Figure 3:**
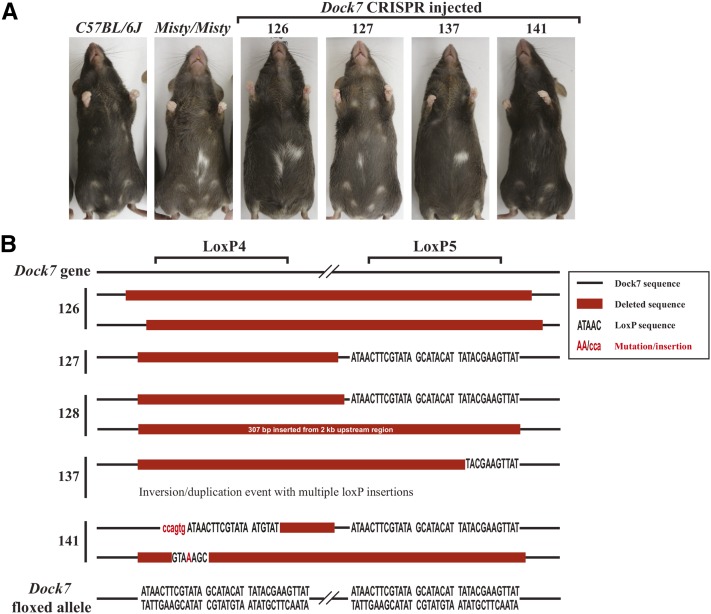
Phenotype and genotyping of *Dock7* null alleles in the cKO1 model. (A) A proportion of mice from the *Dock7* CRISPR/Cas9 injection displayed a diluted coat color and white belly spot similar to that of the *Misty/Misty* mice, which have a no detectable Dock7 protein. This phenotype was found in five out of the 35 mice pups assessed for coat color in the *Dock7* cKO1-2 injection. (B) Summary of sequencing results from mice with diluted coat color and white belly spot from injection *Dock7* cKO1-2. Deletion of exons 3 and 4 was detected by amplifying DNA isolated from mice in (A) and the amplified products were cloned and sequenced, and the sequencing results are depicted in the schematic above. In all five mice, two deletion products were identified, and correspond to the deletion of exons 3 and 4 of *Dock7* gene, similar to what is observed in Figure 4. DNA was amplified with loxP4A F/loxP5A R and sequenced with loxP4B F and/or loxP5B R.

**Figure 4:**
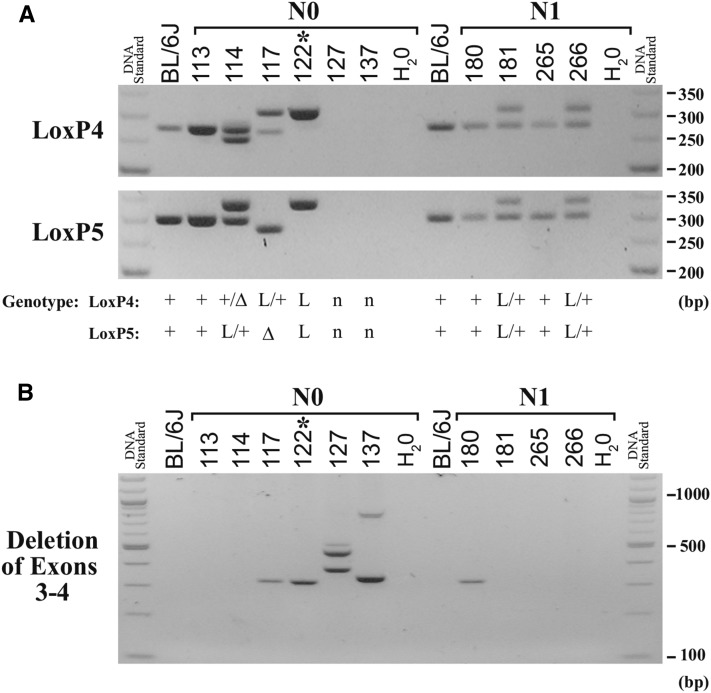
Genotyping of the *Dock7* cKO1 allele. Examples of genotyping are shown for the *Dock7* cKO1 allele. N0 mice were generated from the cKO1-2 CRISPR/Cas9 injections. N1 mice resulted from breeding of 122 female founder, designated with (*) to a C57BL/6J male. DNA ladder size is indicated in base pairs (bp). DNA fragment size of the loxP (L), wild type (+), and small deletions due to Cas9 cleavage (Δ) are indicated. (A) The loxP4 (loxP4B F/R) and loxP5 (loxP5B F/R), and (B) deletion of exons 3–4 (loxP4B F / loxP5B R) primer pairs, were used in genotyping. The corresponding genotypes of the N0 and N1 mice are shown in (A), where (n) indicates no band during genotyping and a potential null allele.

Using the CRISPR/Cas9 system as an alternative method to insert loxP sites within the *Dock7* locus, two conditional deletion strategies were designed (Figure 2). One inserted loxP sites to flank exons 3 and 4 (Figure 2A, cKO1), and the second inserted loxP sites to flank exons 3 and 7 (Figure 2B, cKO2). The loxP insertion sites in the *Dock7* cKO1 model were located within the same introns as the *Dock7^tm1a^* clone, and were the primary focus of this study. Cas9 mRNA, validated sgRNAs, and ∼195 bp oligonucleotide donors containing loxP sites were coinjected into single-cell C57BL/6J zygotes. The resultant mice were genotyped for the presence of the loxP sites, and assessed for phenotypes associated with biallelic disruption of the *Dock7* gene in *Misty* mice (Table 3). Previously, a spontaneous mutant mouse, *Misty*, was identified with a mutation in the *Dock7* allele. Mice homozygous for the *Misty* mutation have undetectable levels of the Dock7 protein (Motyl *et al.* 2013), a diluted coat color, and a white belly spot (Sviderskaya *et al.* 1998) (Figure 3A), which allowed us to easily phenotype potential homozygous null *Dock7* alleles that could have been generated by NHEJ, or incorrect targeting. Biallelic disruption of the *Dock7* gene as indicated by a diluted coat color and a white belly spot was observed in five mice (5/41, 12%) in the cKO1 model (Figure 3A and Table 3). Interestingly, each of the five mice displaying a diluted coat color phenotype had a complete absence of amplified product for both loxP insertion sites (Figure 4A, mouse 127 and mouse 137). To understand the nature of these disruptions, we selected mice with a white belly spot, and genotyped for deletion of the targeted exons with primers flanking exons 3 and 4 as depicted in Figure 2. Each of the five mice genotyped positive for a deletion of DNA, examples are shown in Figure 4B. The amplified product deletion products were sequenced, and the results are diagramed in Figure 3B. While we found evidence for NHEJ (mouse 126), all others had evidence of some insertion from the oligonucleotide, although mutations or deletions were also noted. Furthermore, these mice showed deletion of exons 3 and 4, likely causing the diluted coat color and white belly spot observed in these mice. Because our focus was on generating a conditional, floxed allele, we did not breed mice containing biallelic disruption of the *Dock7* gene to evaluate germline transmission. We then assessed the potential deletion of the DNA between the potential Cas9 cleavage sites located at the loxP4 and loxP5 insertion sites. In our N0 cohort, 45% (19/42) of the mice had some variant of DNA deletion between the two Cas9 cleavage sites (Table 3) as observed in Figure 4B. The disruption of the target gene was found to be a frequent event with CRISPR/Cas9-mediated insertions.

**Table 3:** Frequency of loxP insertion at the *Dock7* locus using CRISPR/Cas

Target Site	Injection Type	Embryos Transferred	Pups Born/Birthrate	Nontransgenic	Loxp4	Loxp5	Both Loxp4 and Loxp5	Deletion Between Target Sites	Null Coat Color
*Dock7* cKO1-1	Pronuclear	150	8 (5%)	4/8 (50%)	2/8 (25%)	4/8 (50%)	2/8 (25%)	4/6 (67%)	0/8 (0%)
*Dock7* cKO1-2	Cytoplasmic	155	39 (25%)	27/39 (69%)	8/39 (21%)	7/39 (18%)	3/39 (8%)	15/35 (42%)	5/35 (14%)

The loxP sites described in the *Dock7* cKO1 model, shown in Figure 2, were introduced by either pronuclear or cytoplasmic injection. Mice were screened by genotyping for loxP4, loxP5, deletion of DNA between the Cas9 cut sites (deletion of exon 3–4), and coat color. The number of mice with the indicated genotype or phenotype is listed compared to the total number of mice analyzed. The percentage of mice with the indicated genotype or phenotype is listed in parentheses. Null coat color indicates diluted coat color with the presence of a white belly spot. An example of genotyping results is shown in in Figure 4. Amplification of DNA from tail/toe clips was performed using primer pairs for loxP4 (loxP4A F/R or loxP4B F/R, which provided identical results and could be used interchangeably), loxP5 (loxP5A F/R or loxP5B F/R for loxP5, which provided identical results and could be used interchangeably), and deletion of exons 3–4 (cKO1 ΔB F/R).

Genotyping was also performed for all N0 mice to detect the loxP site insertions. Examples of genotyping are shown in Figure 4A. The live-birth rate of the injected embryos was 15% (47/305) for the cKO1 model (Table 3). LoxP4 insertions were observed in 10/47 mice (21%), loxP5 insertions were present in 11/47 mice (23%), and insertion of both loxP4 and loxP5 occurred in 11% (5/47) of the N0 mice from the *Dock7* cKO1-1,-2 injection (Table 3). Interestingly, founder 117 not only genotyped positive for the wildtype allele and loxP4 insertion, but also had evidence of deletion of exons 3 and 4 (Table 4). The presence of three or more alleles, including the deletion of exons 3 and 4, for loxP4 or loxP5 was identified in a total of seven (17%) of the 42 N0 mice genotyped, suggesting that Cas9 cleavage is either inducing major recombination events, or the generation of mosaic mice. Nine N0 founder mice with identified loxP sites were bred, and the N1 generation was analyzed by genotyping and sequencing. Overall germline transmission rates were over 89% (8/9) for the mice bred. Offspring number (N1 generation) and genotypes are recorded in Table 4. However, the rate at which loxP sites in the N0 mice were passed to N1 mice varied, and was consistent with mosaicism in the founder mouse. Evidence of mosaicism was also present in *Dock7* cKO1 founder 122, as offspring consisted of mice with floxed alleles as well as a single loxP site. Additionally, five N0 mice, where loxP sites were not detected by genotyping, were bred. For the mice, one to two litters, totaling five to 10 pups, were genotyped, and no mice containing loxP sites were found in the N1 generation (data not shown). While mosaicism could potentially create germline transmission of a loxP site to N1 generation, even though they were not detected in the N0 mice, this scenario was not observed in our cohort, suggesting it is not a frequent event.

**Table 4:** Germline transmission rate of loxP sites in the *Dock7* cKO1 model

	N0 Generation				N1 Generation		
		Genotype			Genotype		LoxP Location
Injection	Mouse ID#	LoxP4	LoxP5	Percentage of Pups with Genotype	LoxP4	LoxP5	(*cis*/*trans*)
cKO1-1	3	L,+	L,+	16/47 (34%)^m^	L*,+	L^m^,+	*cis*
cKO1-2	114	+	L,+	1/4 (25%)	+	L,+	
	116	L	L	18/37 (49%)^m^	L^m^,+	L^m^,+	*cis*
	117	L,+	+	3/27 (11%)*	L*,+	+	
	120	+	L	2/5 (40%)*	+	L*,+	
	122	L	L	9/22 (41%)*	L*,+*^a^*	L*,+*^a^*	*cis**^a^*
				2/22 (9%)	+	L*,+	
				1/22 (5%)	L*,+	+	
	139	L	n	13/27 (48%)*	L*,+	+	
	142	L,+	L	0/28 (0%)	+	+	not tested
	143	+	L	14/36 (39%)*	+	L*,+	

N0 mice from the *Dock7* cKO1 model with one or two loxP sites were bred and the N1 generation was assessed for the floxed allele and germline transmission of the loxP sites. Genotype is indicated by (L) loxP site, (+) wild type, and (n) to indicate no genotyping band and likely a null allele. (*) Represents mice with loxP sites containing correct sequence and (^m^) represent mice with a mutated loxP sequence. Amplification of DNA from tail/toe clips was performed using primer pairs loxP4A F/R or loxP4B F/R for loxP4 which provided identical results and could be used interchangeably. Amplification of DNA from tail/toe clips was formed using primer pairs loxP5A F/R or loxP5B F/R for loxP5 which provided identical results and could be used interchangeably.

a Mice containing the correctly targeted floxed allele.

In a separate set of cytoplasmic injections for the *Dock7* cKO2 model, similar results were observed compared to the *Dock7* cKO1 model. The live-birth rate for transferred embryos was 12%, with 20/174 zygotes resulting in pups. LoxP4 insertions were observed in 7/20 mice (35%), loxP6 insertions were present in 3/20 mice (15%), and insertion of both loxP4 and loxP6 occurred in 1/20 mice (5%) from the *Dock7* cKO2 injection (Table S8). Germline transmission of loxP sites occurred in 5/5 (100%) of the mice bred, and mosaicism was detected in one founder mouse that produced offspring containing floxed alleles or a single loxP site (Table S10). Biallelic disruption of the *Dock7* gene occurred in 5/19 mice assessed for coat color, and deletion of DNA between the Cas9 cut sites was present in 10/20 mice (50%). Examples of genotyping for both loxP insertion and deletion of DNA between the Cas9 cut sites are shown in Figure S2. While the results were overall similar to those observed in the *Dock7* cKO1 model, an increase in biallelic disruption of the *Dock7* gene was observed. It is unclear whether this was due to location of injection (cytoplasmic *vs.* pronuclear), variation between injection days, or was affected by the distance between the Cas9 cleavage sites. Further studies are necessary to investigate these questions.

**Figure 5:**
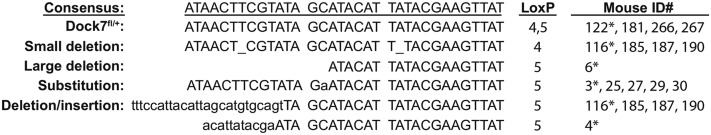
Mutations at loxP insertion sites. LoxP sites in the *Dock7* cKO1 line were sequenced in the N1 and selected N0 mice. Several deviations from the loxP consensus sequence were observed in these mice. The loxP sequence is shown in capital letters, and inserted DNA is shown in lowercase letters. Amplification of isolated DNA was performed using loxP4 (loxP4A F/R) and loxP5 (loxP5A F/R) primers pairs. LoxP4 (loxP4B F/R) and loxP5 (loxP5B F/R) primers were used for sequencing. LoxP mutations in mice 3 and 116 were passed via germline transmission. Mice 4 and 6 were not bred.

The inserted loxP sites and flanking sequences in the N1 and selected N0 mice were cloned and sequenced. Several of the loxP sites sequenced contained small 1- to 2-bp deletions or 1-bp substitutions, whereas some other insertions carried larger deletions within the loxP site itself (Figure 5). Correctly sequenced and mutated loxP sites are indicated in Table 4 and Table S10 for the cKO1 and cKO2 models, respectively. From either model, only founders 122 (cKO1) and 17 (cKO2) produced floxed *Dock7* mice, with two correctly sequenced loxP sites in *cis* orientation. These loxP mutations were observed in N0, and their respective offspring. In order to determine if any of these mutations of the insertion sequence were introduced during oligonucleotide synthesis, the oligonucleotide donors for loxP4 and loxP5 were cloned and sequenced. The oligonucleotide donors indeed contained 1- to 2-bp deletions and a 1-bp substitution within the loxP site and/or surrounding sequence (Table 5). These mutations observed in the oligonucleotide donors were similar to the type of mutation observed in the CRISPR-generated transgenic mice, suggesting some of the mutations occurred during the synthesis process. However, no large deletions or insertions in the loxP site were observed in the oligonucleotide donors. These data suggest that some of the small mutations in the oligonucleotides may be responsible for similar mutations observed in the inserted loxP sites. We believe that the larger deletions or insertions of DNA sequences occurred during the recombination process.

**Table 5:** Sequence analysis of oligonucleotide donors

Oligonucleotide	30 bp Upstream	LoxP Sequence	30 bp Downstream
LoxP4 oligonucleotide	TCCATTACATTAGCATGTGCAGTGGCCATG	ATAACTTCGTATAGCATACATTATACGAAGTTAT	GTCAGGGTGTGGAGCGTTTTGGGAGCTTTA
	TCCATTACATTAGCATGTGCAGTGGCCATG	ATAACTTCGTATAGCATACATTATACGAAGTTAT	GTCAGGGTGTGGAGCGTTTTGGGAGCTTTA
	TCCATTACATTAGCATGTGCAGTGGCCATG	ATAACTTCGTATAGCATACATTATACGAAGTTAT	GTCAGGGTGTGGAGCGTTTTGGGAGCTTTA
	TCCATTACATTAGCATGTGCAGTGGCCATG	ATAA__TCGTATAGCATACATTATACGAAGTTAT	GTCAGGGTGTGGAGCGTTTTGGGAGCTTTA
	TCCATTACAT_AGCATGTGCAGTGGCCATG	ATAACTTCGTATAGCATACATTATACGAAGTTAT	GTCAGGGTGTGGAGCGTTT_GGGAGCTTTA
	TCCATTACATTAGCATGTGCAGTGGCCATG	_TAACTTCGTATAGCATACATTATACGAAGTTAT	GTCAGGGTGTGGAGCGTTTTGGGAGCTTTA
	TCCATTACATTAGCATGTGCAGTGGCCATG	ATAACTTCGTATAGCATACATTATACGAAGT_AT	GTCAGGGTGTGGAGCGTTTTGGGAGCTTTA
	TCCATTACATTAGCATGTGCAGTGGCCATG	ATAACTTCGTATAGCATACATTATACGAAGTTAT	GTCAGGGTGTGGAGCGTTTTGGGAGCTTTA
	TCCATTACATTAGCATGTGCAGTGGCCATG	ATAACTTCGTATAGCATACATTATACGAAGTTAT	GTCAGGGTGTGGAGCGTTTTGGGAGCTTTA
	TCCATTACATTAGCATGTGCAGTGGCCATG	ATAACTTCGTATAGCATACATTATACGAAGTTAT	GTCAGGGTGTGGAGCGTTTTGGGAGCTTTA
LoxP5 oligonucleotide	TTGCAGAGGACCTGGGTTTGGTTCCTAGCT	ATAACTTCGTATAG_ATACATTATACGAAGTTAT	CACTGGCATCTAACAGCCATCTCTACCTCC
	TTGCAGAGGACCTGGGTTTGGTTCCTAGCT	ATAACTTCGTATAGCATACATTATACGAAGTTAT	CACTGGCATCTAACAGCCATCTCTACCTCC
	TTGCAGAGGACCTGGGTTTGGTTCCTAGCT	ATAACTTCGTATAGCATACATTATACGAAGTTAT	CACTGGCATCTAACAGCCATCTCTACCTCC
	TTGCAGAGGAC_TGGGTTTGGTTCCTAGCT	ATAACTTCGTATAGCATACATTATACGAAGTTAT	CACTGGCATCTAACAGCCATCTCTACCTCC
	TTGCAGAGGACCTGGGTTTGGTTCCTAGCT	ATAACTTCGTATAGCATACATTATACGAAGTTAT	CACTGGCATCTAACAGCCATCTCTACCTCC
	TTGCAGAGGACCTGGGTTTGGTTCCTAGCT	ATAACTTCGTATAGCATACATTATACGAAGTTAT	CACTGGCATCTAACAGCCATCTCTACCTCC
	TTGCAGAGGACCTGGGTTTGGTTCCTAGCT	ATAACTTCGTATAGCATACATTATACGAAGTTAT	CACTGGCATCTAACAGCCATCTCTACCTCC
	TTGCAGAGGACCTGGGTTTGGTTCCTAGCT	ATAACTTCGTATAGCATACATTATACGAAGTTAT	CACTGGCATCTAACAGCCATCTCTACCTCC
	TTGCAGAGGACCTGGGTTTGGTTCCTAGCT	ATAACTTCGTATAGCATACATTATACGAAGTTAT	CACTGGCACCTAACAGCCATCTCTACCTCC

Both loxP4 (194 bp, top) and loxP5 (193 bp, bottom) oligonucleotide donors were cloned, and 10 clones of loxP4 oligo donor and nine clones of loxP5 donor were sequenced. A series of 1- or 2-bp deletions were identified in both oligonucleotides with a 1-bp substitution was present in the loxp5 oligonucleotide. Observed mutation or substitutions are shown in gray.

**Figure 6:**
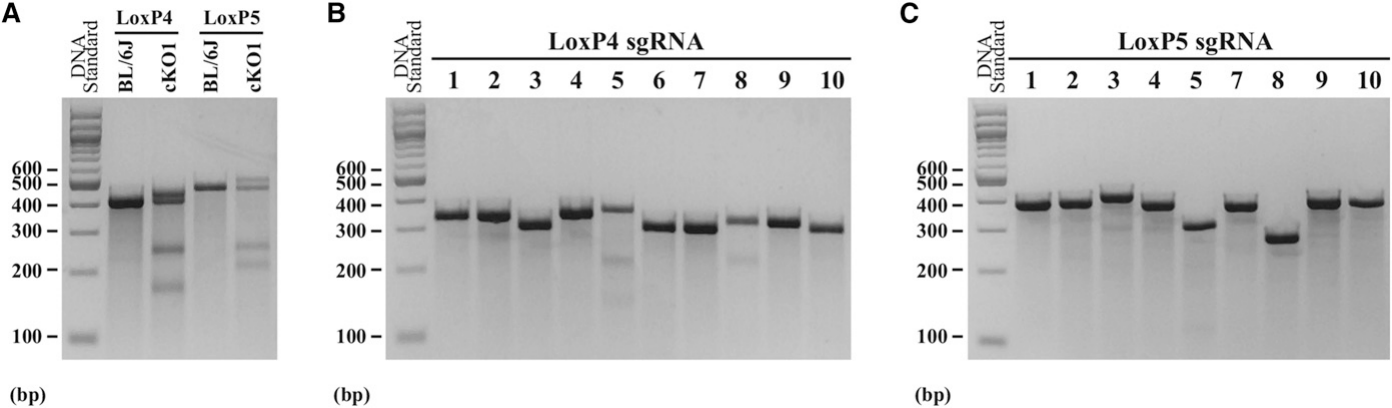
Analysis of potential off-target effects in the *Dock7* cKO1. The SURVEYOR mutation assay was used to evaluate off-target cleavage by Cas9. (A) LoxP4 and loxP5 insertion sites were used as a positive control. Cas9 activity was screened at the top 10 homologous sites in the genome to the sgRNA for both loxP4 (B) and loxP5 (C) in the N0 mouse, using primer pairs listed in Table S5 and Table S6. As a negative control, off-target activity was assessed in the wild-type C57BL/6J mouse at the insertion sites for both loxP4 and loxP6, as shown in Figure S4. Primer pairs for loxP4 (loxP4A F/R) and loxP5 (loxP5B F/R) were used for amplification of control DNA samples.

Sequencing of the *Dock7* locus in founders 122 (cKO1) and 17 (cKO2) confirmed correct insertion of both loxP sites creating the floxed *Dock7* allele. Only these mice produced offspring containing two loxP sites on the same chromosome with the correct sequence. To calculate the success rate in generating the *Dock7* floxed alleles, we divided the number of correctly targeted founders by the total number of embryos transferred per cKO model. This represents a 0.33% (1/305) and 0.57% (1/174) success rate for the models, respectively, for the *Dock7* cKO1 and cKO2 models (Table S9). Only 2–5% of pups born were capable of establishing a mouse line bearing the correct floxed allele. In order to test off-target Cas9 cleavage activity, the top 10 potential off-target sites were identified as predicted by the algorithm from the Zhang lab, and the cleavage activity at these sites were tested using the SURVEYOR mutation assay. No off-target activity was observed for either the *Dock7* cKO1 (Figure 6) or the *Dock7* cKO2 (Figure S3) model. DNA isolated from C57BL/6J mice was used as a negative control (Figure S4).

As a phenotypic method to analyze the *Dock7* cKO1 model, and to assess the functionality of the Dock floxed allele, we crossed the *Dock7* cKO1 model with the Sox2-Cre strain, which expresses Cre protein in the early germline and is efficient in generating germline deletions. Following Cre recombination, excision of exons 3 and 4 was confirmed by DNA sequencing (data not shown). Mice homozygous for the *Dock7* null allele (*Dock7*^−/−^) generated through Cre recombination showed similar coat color and white belly (Figure S5) as the *Misty* mice and *Dock7* null mice generated through biallelic disruption of the *Dock7* gene by CRISPR/Cas (Figure 3). These data confirm that the floxed allele can undergo Cre recombination, that the coat color phenotype is a direct result of disruption of the *Dock7* gene, and that deletion of exon 3 and 4 in the *Dock7* gene is sufficient to elicit the null *Dock7* phenotype.

## Discussion

The establishment of the KOMP, a resource for targeted C57BL/6N ES cell lines, has dramatically enhanced both the speed and quality of research on the C57BL/6 inbred background (Bradley *et al.* 2012). However, one limitation is the less than ideal success rate of germline transmission of the targeted ES cell lines. Interestingly, even with a small group of six targeted alleles, the success rate observed in this study recapitulated that observed by the KOMP repository of ∼50% [KOMP repository germline transmission rates and (Cotton *et al.* 2015)]. Furthermore, additional issues that prevented generation of the *Dock7* transgenic line from the KOMP-targeted cells included an incomplete targeting cassette and a mixed population of ES cells from the parental “clone” (Ryder *et al.* 2013). Our observations indicate that characterization of the targeted ES cell clones prior to injection is an important validation step.

The development and application of the CRISPR/Cas9 system was a major advancement in the generation of genetically modified mice. The recent successful application of CRISPR/Cas9 technology in genome modification, regardless of species and genetic background, has established it as an efficient and powerful alternative to traditional gene targeting. It is now possible to generate an NHEJ-mediated gene disruption in mice in as little as 4 wk. However, the efficiency of targeted genomic insertion was at least two-fold lower than that of deletions (Wang *et al.* 2013; Yang *et al.* 2013). The rate at which both loxP sites were incorporated ranged from 5/47 (11%) for cKO1 to 1/20 (5%) cKO2 in our models. However, the rate at which a correctly sequenced floxed allele in cKO1 was generated and passed to the N1 generation was lower, at 1/47 (2%). Of the four additional mice that showed evidence of insertion of loxP4 and loxP5, two were found to have mutated loxP sites, one died prior to weaning, and the final mouse had no germline transmission of either loxP site after four litters.

One of the benefits to CRISPR/Cas9 is that the genome of any mouse strain can be manipulated. Previous studies have demonstrated success in hybrid/outbred strains (Wang *et al.* 2013; Yang *et al.* 2013), and mice in a C57BL/6N background (Lee and Lloyd 2014; Yen *et al.* 2014). However, it is still unclear if variability between mouse strains can affect the overall outcome of success in developing a floxed allele using CRISPR/Cas9. Since we generated only one functional floxed *Dock7* mouse in each model using C57BL/6J zygotes, our data suggest that that injection and transfer of at least 300–400 C57BL/6J zygotes may provide the necessary coverage to generate between 40 and 60 N0 mice in the C57BL/6J background, which is a reasonable number to screen.

Genomic manipulation by CRISPR/Cas9 has been associated with robust rates of germline transmission (Li *et al.* 2013a), 2013b). In our study, we chose to primarily breed N0 mice containing either one or two loxP sites. Germline transmission rates of loxP sites were above 93% for the mice bred. Furthermore, germline transmission of loxP sites was generally achieved within one to two litters, requiring screening an average of seven pups per founder. Only founder 117 required screening of 19 pups (three litters). All other founders required screening of only six pups, or one litter, including both founders harboring the correctly sequenced floxed alleles. Founder 142 did not produce germline transmission of single inserted loxP site after 28 pups or four litters; since founder 142 genotyped positive for only one loxP site, breeding was discontinued. In all cases, no offspring genotyped positive for loxP sites that were not detected in the N0 generation from either transgenic or nontransgenic mice. These data suggest that priority should be placed on screening offspring from transgenic founders positive for both loxP sites, and that identification of potential loxP sites should be reliable in the N0 generation. Furthermore, screening of the N1 generation can be accomplished relatively quickly in one to two litters. However, with the necessity of screening the N1 generation to confirm the germline transmission of the floxed allele, and the potential for mutations in the regions of recombination, the length of time necessary to validate a conditional mouse model can be more extensive than expected. As every genetic locus is different, and loxP insertion frequency is likely to be dependent on the surrounding sequence or transcriptional activity of the gene, there are no standard guidelines that can be extrapolated from targeting one particular gene.

Heterogeneity in the N1 generation due to mosaicism has been observed previously (Yen *et al.* 2014; Yang *et al.* 2013). Mosaicism in the founder mice was present in both founders that produced the correctly targeted *Dock7* cKO1 and cKO2 mice, with variation in the number of loxP sites passed from the N0 to N1 generation. Additional evidence of mosaicism could be observed in the N0 mice containing both a wild-type and loxP positive allele as well as a deletion of DNA between the Cas9 cut sites, such as in the loxP4 genotyping in cKO1 founder 117. Therefore with the frequent occurrence of mosaicism with CRISPR/Cas9, screening the N1 generation is necessary to confirm the establishment of the conditional deletion line.

Due to the novelty of the CRISPR/Cas9 system, many of the technical issues related to the optimal strategy of simultaneous insertion of two loxP sites using two sgRNAs and two oligonucleotide donors have not been established. Various mutant alleles can result from this strategy, including deletions at either or both of the target sites, or between the two target sites by NHEJ, a single targeted loxP site, or two targeted loxP sites. In addition, if both targeted loxP sites were inserted, they could be either on the same or a different allele of the gene if located on an autosome. Our screening of a large cohort of N0 and N1 mice revealed all of the aforementioned varieties of modified alleles, as well as mutated and partial loxP sites. Several loxP variants occurred in mice with two correctly inserted loxP sites, thus eliminating their use as a conditional deletion model, and decreasing the efficiency of generating a correctly targeted allele. Further investigation suggested that, in some cases, 1- to 2-bp mutations in the loxP sites themselves were in fact present in the donor oligonucleotides. Therefore, it is likely that use of oligonucleotides of almost 200 bp greatly increases the error rate. We hypothesize that smaller oligonucleotides, or oligonucleotides generated from a different source, potentially a sequenced plasmid, might decrease the frequency of error and increase the correct targeting allele efficiency. However, it is unclear if the partial loxP sites observed here are direct results of a partial insertion or of full insertion followed by a subsequent deletion of DNA within the loxP site. Further work is necessary to identify the mechanisms of DNA deletion and mutation observed in loxP sequences.

Among the mice we obtained from zygote injections, deletion of genomic DNA between the Cas9 cut sites occurred in 42–67% of mice. This was 1.5- to 2-fold higher than the loxP site insertion rate (18–50%), which is similar to what has been previously reported (Yang *et al.* 2013), suggesting that NHEJ is still more efficient than homologous recombination. We also observed large biallelic deletions in between the Cas9 cut sites at a rate between 12% and 25% among mice generated from injected zygotes. The use of a visible phenotype greatly enhanced the screening process for these alleles. Although mice with this type of DNA deletion may in fact be a useful complement to conditional null alleles, production of these alleles still reduced the overall efficiency of generation of the desired transgenic strains. As deletion of DNA between the Cas9 cut sites occurred in all three injections at varying rates, it is unclear what variables may increase the rate of these deletions, including the location of injection (pronuclear *vs.* cytoplasmic), the distance between Cas9 cut sites, and gene-to-gene variability. SCR7—a DNA ligase IV inhibitor—has been used as an inhibitor of NHEJ to promote homology-mediated repair (Vartak and Raghavan 2015), but that strategy was not utilized in this study. Lastly, it is worth noting that the use of CRISPR/Cas9 to generate conditional deletion mice of heterozygous or homozygous lethal genes would naturally select against embryos containing these global deletion alleles, thus resulting in fewer N0 mice, but increasing targeting efficiency results.

Overall, we succeeded in the generation of two novel, conditional, null alleles of the *Dock7* gene using the CRISPR/Cas9 system to target two intronic sites. These sites were modified with loxP sequences provided via oligonucleotide donors. Our results showed a greater than expected variability in sequence within and surrounding the loxP sites, highlighting the importance of full characterization of the modified sequence.

## Supplementary Material

Supplemental Material
