# Supplementary material for: CRISPR/Cas9-Mediated Insertion of loxP Sites in the Mouse Dock7 Gene Provides an Effective Alternative to Use of Targeted Embryonic Stem Cells
Source: G3 (Bethesda). 2016 May 11;6(7):2051–61. doi: 10.1534/g3.116.030601 (PMC4938658; doi:10.1534/g3.116.030601)
Supplement: Supplemental Material [file supp_6_7_2051__index.html]

CRISPR/Cas9-Mediated Insertion of loxP Sites in the Mouse Dock7 Gene Provides an Effective Alternative to Use of Targeted Embryonic Stem Cells — Supplemental Material 

# CRISPR/Cas9-Mediated Insertion of loxP Sites in the Mouse *Dock7* Gene Provides an Effective Alternative to Use of Targeted Embryonic Stem Cells

## Supplemental Material for Bishop *et al.*, 2016

**Files in this Data Supplement:**

- Table S1 - *Dock7tmla* genotyping primer pairs. (.pdf, 107 KB)
- Table S10 - Germline transmission rate of loxP sites in the *Dock7* cKO2 model. (.pdf, 16 KB)
- Table S2 - Cycling condition and reaction components for genotyping and cloning. (.pdf, 15 KB)
- Table S3 - *Dock7* CRISPR reagents. (.pdf, 105 KB)
- Table S4 - *Dock7* CRISPR primer pairs. (.pdf, 219 KB)
- Table S5 - Primer pairs to assess off target (ot) Cas9 activity during CRISPR-Cas9 insertion of loxP4. (.pdf, 110 KB)
- Table S6 - Primer pairs to assess off target (ot) Cas9 activity during CRISPR-Cas9 insertion of loxP5. (.pdf, 110 KB)
- Table S7 - Primer pairs to assess off target (ot) Cas9 activity during CRISPR-Cas9 insertion of loxP6. (.pdf, 111 KB)
- Table S8 - Frequency of CRISPR-Cas9-mediated loxP insertion in the *Dock7* cKO2 model. (.pdf, 117 KB)
- Table S9 - Frequency of CRISPR-Cas9-mediated loxP insertion in the *Dock7* locus. (.pdf, 109 KB)
- Figure S1 - Validation of sgRNAs. (.eps, 6381 KB)
- Figure S2 - Genotyping of the *Dock7* cKO2 allele. (.eps, 1613 KB)
- Figure S3 - Analysis of potential off target effects in the *Dock7* cKO2. (.eps, 1473 KB)
- Figure S4 - C57BL/6J mice as a negative control for SURVEYOR mutation assay. (.eps, 1484 KB)
- Figure S5 - Phenotype of the *Dock7*-/- generated by Cre recombination. (.eps, 3217 KB)
