# Supplementary material for: CRISPR/Cas9-Mediated Insertion of loxP Sites in the Mouse Dock7 Gene Provides an Effective Alternative to Use of Targeted Embryonic Stem Cells
Source: G3 (Bethesda). 2016 May 11;6(7):2051–61. doi: 10.1534/g3.116.030601 (PMC4938658; doi:10.1534/g3.116.030601)
Supplement: Supplemental Material [file supp_g3.116.030601_TableS1.pdf]

Primer pair	Forward (5’-3’)	Reverse (5’-3’)
Neo-loxP2A	TCCCCCTGAACCTGAAACATAA	TCATTTTATCCGCCTACTGCGA
Neo-loxP2B	CTCCCCCTGAACCTGAAACATAAA	CTCGAAACCCTCACGTACAGAC
LoxP3	CATTATACGAAGTTATGGTCTGAGC	AGAAGAACACGAGAACGCCTTT
PCR Control	TGTTGGTGTCAGAGAAAGTTGGC	CCTCGGGGGAAAGCACTG

Table S1. ***Dock7***^{tmla} **genotyping primer pairs.** Sequences of primers used for genotyping of the *Dock7*^{tm1a} cassette are listed. Forward (F) and reverse (R) primers are abbreviated accordingly. The PCR control is located in *Dock7* exon 18, approximately 68 kb downstream of the cassette insertion. All primer pairs were used with the MasterTaq thermocycling protocol described in Table S2.
