# Supplementary material for: CRISPR/Cas9-Mediated Insertion of loxP Sites in the Mouse Dock7 Gene Provides an Effective Alternative to Use of Targeted Embryonic Stem Cells
Source: G3 (Bethesda). 2016 May 11;6(7):2051–61. doi: 10.1534/g3.116.030601 (PMC4938658; doi:10.1534/g3.116.030601)
Supplement: Supplemental Material [file supp_g3.116.030601_TableS10.pdf]

	N0 Generation				N1 Generation		
Injection	Mouse ID#	Genotype		Percentage of pups with genotype	Genotype		LoxP orientation
		LoxP4	LoxP6		LoxP4	LoxP6	(cis/trans)
cKO2	3	L,+	+	4/15 (27%)*	L*,+	+	
	4	L,+	+	1/2 (50%)	L,+	+	
	10	+	L	34/34 (100%)	+	L/+	
	13	L,+	+	15/29 (52%)*	L*,+	+	
	17	L	L	11/29 (38%)*	**L*,+**	**L/+**	Cis
				3/29 (10%)	L,+	+	

Table S10. **Germline transmission rate of loxP sites in the *Dock7* cKO2 model.** N0 mice from the *Dock7* cKO2 injection with one or two loxP sites were bred and the N1 generation was assessed for the floxed allele and germline transmission of the loxP sites. Genotype is indicated by (L) loxP site, (+) wild type, and (n) to indicate no genotyping band and likely a null allele. (*) Represents mice with loxP sites containing correct sequence and (m) represent mice with a mutated or truncated loxP sequence. The mice containing the correctly targeted floxed allele are bolded in the far right column. Amplification of DNA from tail/toe clips was preformed using primer pairs loxP4A F/R or loxP4B F/R for loxP4 which provided identical results and could be used interchangeably. Amplification of DNA from tail/toe clips was formed using primer pair loxP6A F/R.
