# Supplementary material for: CRISPR/Cas9-Mediated Insertion of loxP Sites in the Mouse Dock7 Gene Provides an Effective Alternative to Use of Targeted Embryonic Stem Cells
Source: G3 (Bethesda). 2016 May 11;6(7):2051–61. doi: 10.1534/g3.116.030601 (PMC4938658; doi:10.1534/g3.116.030601)
Supplement: Supplemental Material [file supp_g3.116.030601_TableS2.pdf]

	**MasterTaq (5 prime)**	**AccuStart II PCR SuperMix (Quanta Biosciences)**	**JumpStart Taq (Sigma)**	**Terra Taq (Clontech)**	**Phusion Polymerase (NEB)**
Cycling	1. 97ºC; 30 sec<br>2. 94ºC; 15 sec<br>3. 55ºC; 30 sec<br>4. 72ºC; 30 sec<br>+1 sec/cycle<br>39 cycles (steps 2-4)<br>5. 72ºC; 10 min	1. 95ºC; 2 min<br>2. 95ºC; 15 sec<br>3. 60ºC; 15 sec<br>4. 72ºC; 20 sec<br>35 cycles (steps 2-4)	1. 95ºC; 5 min<br>2. 95ºC; 30 sec<br>3. 60ºC; 30 sec<br>4. 68ºC; 40 sec<br>5. cycles (steps 2-4)<br>6. 68ºC; 5 min	1. 98ºC; 2 min<br>2. 98ºC; 10 sec<br>3. 60ºC; 15 sec<br>4. 68ºC; 1 min<br>26 cycles (steps 2-4)	1. 98ºC; 30 sec<br>2. 98ºC; 10 sec<br>3. 60ºC*; 30 sec<br>4. 72ºC; 30 sec<br>39 cycles (steps 2-4)<br>5. 72ºC; 10 min<br><br>*Annealing temp of 55ºC was used for oligo cloning
PCR reaction	ddH_20 16.4 µL<br>10x Buffer 2.5 µL<br>5 mM dNTPs 1.0 µL<br>10 µM F primer 1.0 µL<br>10 µM R primer 1.0 µL<br>DNA 3.0 µL<br>Polymerase 0.10 µL	ddH_20 6.5 µL<br>2x Mix 12.5 µL<br>10 µM F primer 2.5 µL<br>10 µM R primer 2.5 µL<br>DNA 1.0 µL	ddH_20 6.5 µL<br>2x Mix 12.5 µL<br>10 µM F primer 2.5 µL<br>10 µM R primer 2.5 µL<br>DNA 1.0 µL	ddH_20 9.5 µL<br>2x Buffer 12.5 µL<br>10 µM F primer 0.75 µL<br>10 µM R primer 0.75 µL<br>DNA 1.0 µL<br>Taq 0.5 µL	ddH_20 14.25 µL<br>5x Buffer 5.0 µL<br>5 mM dNTPs 1.0 µL<br>10 µM F primer 1.25 µL<br>10 µM R primer 1.25 µL<br>DNA or 2.0 µL<br>10 ng oligo<br>Polymerase 0.25 µL

Table S2. **Cycling condition and reaction components for genotyping and cloning.**
