# Supplementary material for: CRISPR/Cas9-Mediated Insertion of loxP Sites in the Mouse Dock7 Gene Provides an Effective Alternative to Use of Targeted Embryonic Stem Cells
Source: G3 (Bethesda). 2016 May 11;6(7):2051–61. doi: 10.1534/g3.116.030601 (PMC4938658; doi:10.1534/g3.116.030601)
Supplement: Supplemental Material [file supp_g3.116.030601_TableS3.pdf]

LoxP site:	sgRNA target recognition sites with genomic coordinates	Oligonucleotide (w/loxP site)
LoxP4	acgctccacaccctgacccatgg<br><br>chr4:+98750272	tccatcttgggccttttaaccatgtgcaaagactctccctctactttatttccattacattagcatgtgcagtggccatgataacttcgtatagcatacattatacgaagttatgtcagggtgtggagcgttttgggagctttacctgccacagtctggtcacagtatttgctagaatgatccccagtaacaac
LoxP5	gggtttggttcctagctacactgg<br><br>chr4:-98748262	agtgatgtggggctggagaggaggctctagtttagagcactggctattcttgcagaggacctgggtttggttcctagctataacttcgtatagcatacattatacgaagttatcactggcatctaacagccatctctacctcctgatccaggtatctgacatcctcttctgtgagcagtgcatgcacatggtg
LoxP6	ccaaccattgagctagcttaggg<br><br>chr4:+98744990	ttccagacaccccaatgctctggagtccccacataaaaggcacaaatgtacagccttatttttaatataccctaaataacttcgtatagcatacattatacgaagttatggatccaatggttgggcacttccaaacctcctcacagctagcacacactcccctccagtatacctgagttaacactttcta

Table S3. ***Dock7*** **CRISPR reagents.** Sequences of the sgRNA, sgRNA genomic coordinates, and the sequence of oligonucleotides loxP donors are listed. The loxP site is underlined in the oligonucleotide. Genomic coordinates are expressed in the NCBI37/mm9 build.
