# Supplementary material for: CRISPR/Cas9-Mediated Insertion of loxP Sites in the Mouse Dock7 Gene Provides an Effective Alternative to Use of Targeted Embryonic Stem Cells
Source: G3 (Bethesda). 2016 May 11;6(7):2051–61. doi: 10.1534/g3.116.030601 (PMC4938658; doi:10.1534/g3.116.030601)
Supplement: Supplemental Material [file supp_g3.116.030601_TableS4.pdf]

	Anaysis					PCR		
**Primer pair**	**G**	**C**	**S**	**Forward (5'-3')**	**Reverse (5'-3')**	**J**	**T**	**P**
LoxP4A	x	x		CCAGCGTACACAGCCCAGATCA	CGAAACCCTCACGTACAGACTCTCC	X	X	
LoxP4B	x		x	GCCCAGATCACCCTTGTCTC	TGCTAAGTTTCTAAAAGCTGCCT		X	
LoxP5A	x	x		TGAAGAAGGGTCTTCATTCTAGA ACATTT	TCACACTTAAACTAACAAGGTTATCAG GGT	X	X	
LoxP5B	x		x	TTCACTGATGGAATAATCTGTGCT	GCAGCCAAGAGGGCAAAAC		X	
LoxP6A	x	x	x	GTAAATTCTTCCTGGCTGGGGT	TTCCATAGGATGGACCAAGCAG	X	X	
LoxP6B	x			GAGACAAGACTCTTTGTTTCTC	GGTCTTTGATTTGTGCTTACAG	X		
cKO1 ΔA		x		CCAGCGTACACAGCCCAGATCA	TCACACTTAAACTAACAAGGTTATCAG GGT		X	
cKO1 ΔB	x		x	GCCCAGATCACCCTTGTCTC	GCAGCCAAGAGGGCAAAAC		X	
cKO2 Δ	x			GCCCAGATCACCCTTGTCTC	TTCCATAGGATGGACCAAGCAG		X	
Oligo4		x		GGACAGTGTCTCCATCTTGG	CATTTTATCTGTTGTTACTGG			X
Oligo5		x		ATTACTAACATAAGTGATGTGG	TGGAACTCTGTGCACCATGTGC			X
T7 promoter			x	TAATACGACTCACTATAGGG				

Table S4. *Dock7* CRISPR primer pairs. Primer pairs were used to analyze the *Dock7* gene by genotyping (G), cloning (C), and sequencing (S). The cKO1 Δ primer pairs were used to assess deletion of exons 3 and 4 in the *Dock7* gene. The cKO2 Δ primer pairs used to assess deletion of exons 3 through 7 in the *Dock7* gene. The amplification of isolated DNA was performed according to the Jumpstart (J), Terra Taq (T), or Phusion polymerase (P) protocols described in Table S2. Forward (F) and reverse (R) primers are abbreviated accordingly.
