# Supplementary material for: CRISPR/Cas9-Mediated Insertion of loxP Sites in the Mouse Dock7 Gene Provides an Effective Alternative to Use of Targeted Embryonic Stem Cells
Source: G3 (Bethesda). 2016 May 11;6(7):2051–61. doi: 10.1534/g3.116.030601 (PMC4938658; doi:10.1534/g3.116.030601)
Supplement: Supplemental Material [file supp_g3.116.030601_TableS5.pdf]

Primer pair	Off target site (NCBI37/mm9)	Forward (5’-3’)	Reverse (5’-3’)
LoxP4ot 1	chr15:+72611911	TCTATTCAAGATTAAGGTACTGGCTGGC	ACTTCATTCACAAAGGCCTGACCA
LoxP4ot 2	chr16:+42168470	ACAGCCATATAGTGTGGTCTCATGCA	GCAACTTGGCAATGGATTTACTTGAAT
LoxP4ot 3	chr7:+28954978	TGAAGCCAGGTAAGGACCACGG	GGGACCAAGGTATCGTTGACAGG
LoxP4ot 4	chr4:+117809504	CTCTTGAGAAGACCGTAGTCCAATCCT	GGAGGGCTTCATACCTTGCTCTGTATA
LoxP4ot 5	chr3:+67813766	TAAGGTACTGGGGGAGGCAGTTCA	TTCCGGGAGCTGAACACAAGTG
LoxP4ot 6	chr4:+138451382	ACTCCTGAGCTGAGAAACCAAGACG	GAGCCAGTGTCCTTCAAGCTGTTTT
LoxP4ot 7	chr16:+35402029	CAATGGCTTCCAGACACCAGTGTAC	GTTCACACCTTCATCCCATTCCC
LoxP4ot 8	chr11:-96986362	TGGGCAGCTGGCATTCTGTG	TAGGGTAGCAGGTGTTCCGAGAGG
LoxP4ot 9	chr16:+67621197	AGATGCCAGCCTTGACACAGGA	GGTGGCGATTGGCTGAATTCTT
LoxP4ot 10	chr11:-34085573	ACTTGATCTATGTCCATGCTAAGAGTGATG	TCCTGTATGCTTTACTGGGGTTCCT

Table S5. Primer pairs to assess off target (ot) Cas9 activity during CRISPR-Cas9 insertion of loxP4. Genomic coordinates for potential off target regions were listed in the NCBI37/mm9 build. These primer pairs were used in SURVEYOR mutation assays. Forward (F) and reverse (R) primers were abbreviated accordingly and cycling conditions using JumpStart Taq can be found in Table S2.
