# Supplementary material for: CRISPR/Cas9-Mediated Insertion of loxP Sites in the Mouse Dock7 Gene Provides an Effective Alternative to Use of Targeted Embryonic Stem Cells
Source: G3 (Bethesda). 2016 May 11;6(7):2051–61. doi: 10.1534/g3.116.030601 (PMC4938658; doi:10.1534/g3.116.030601)
Supplement: Supplemental Material [file supp_g3.116.030601_TableS6.pdf]

Primer pair	Off target site (NCBI37/mm9)	Forward (5'-3')	Reverse (5'-3')
LoxP5o t1	chr1:+6805634	ACTTGATCTATGTCCATGCTAAGAGTGATG	GACAAAATCCAACACCCATTCATGAT
LoxP5ot 2	chr14:+61414767	CTTCAAGCCTGAAGACAGAATGAAAAGA	CAAAAACCAGTCTCTATGTTCAATCAGGAT
LoxP5ot 3	chr3:-95805557	ATCTATTTAGGGACTTTGATGACTCACAGC	CAGTCTATCCATTTTTAGAGCTGGGCA
LoxP5ot 4	chr1:-20708183	ATGTACCATTTAGATGTCTGTCAGGAACAC	GACAGAAACTCAGTTGGGCTAGTCTGA
LoxP5ot 5	chr1:+65426077	TGAACATTGGTTTTGGTTCCCAG	CAGCTCTCAGGACCTACCCATACTTGT
LoxP5ot 6	chr16:-36807690	Too repetitive to design primers	Too repetitive to design primers
LoxP5ot 7	chr5:-100040548	TGCTCCATCTGACAGTCAAGGTAGG	GCAGGCCTGCCTGGAAGTTATG
LoxP5ot 8	chr14:+40469702	CTCTCTCTCTCCTCCCTACCTCTCCAC	GGTAGCCTGCTGGCTAGTATGAGAGG
LoxP5ot 9	chr4:-83614080	GTTCCTTATTTCCCGATTTTCAGAATAGG	AAGTTGCAATGGAGGACATCCCA
LoxP5ot 10	chr11:+113188213	AGTTCCCAAGTGTAAGCTCTTCGGA	TGTGTTTGGCCTCCAGTCTGTCC

Table S6. Primer pairs to assess off target (ot) Cas9 activity during CRISPR-Cas9 insertion of loxP5.

Genomic coordinates for potential off target regions were listed in the NCBI37/mm9 build. These primer pairs were used in SURVEYOR mutation assays. Forward (F) and reverse (R) primers were abbreviated accordingly and cycling conditions using JumpStart Taq can be found in Table S2.
