# Supplementary material for: CRISPR/Cas9-Mediated Insertion of loxP Sites in the Mouse Dock7 Gene Provides an Effective Alternative to Use of Targeted Embryonic Stem Cells
Source: G3 (Bethesda). 2016 May 11;6(7):2051–61. doi: 10.1534/g3.116.030601 (PMC4938658; doi:10.1534/g3.116.030601)
Supplement: Supplemental Material [file supp_g3.116.030601_TableS7.pdf]

**Primer pair**	**Off target site (NCBI37/mm9)**	**Forward (5'-3')**	**Reverse (5'-3')**
LoxP6ot 1	chr7:-60907931	ATTGTCTCCCAAGGAAGAACTC	ACAGTGACCAGAATTAGAGTAT AAGG
LoxP6ot 2	chr6:-28463696	TCTCCCTAGTCCTTCTGTGTAG	AGGCAGGAGGATCAGAAATTG
LoxP6ot 3	chr15:+72473382	CCAATCCATCTTCTGACCTCTC	ATCATGGGCAGGTTCCTTATC
LoxP6ot 4	chr14:-66373202	CCATGGTTGCTGCGTTTAG	GCACTGTTTCTTTCTTCACTCC
LoxP6ot 5	chr9:+79417635	AAATGAAGGGACCTGTCTATGG	AAGTGTTCAGACTTCCCAGTTAT
LoxP6ot 6	chr4:-115312664	CAGTGATGAGGGTCTAAACAGG	GCTTCCATTATGATGTGCTGAAG
LoxP6ot 7	chr9:-64594834	GTCAAAGCAGAGCTCACTAGAA	ATACTACTGCCTCTAGCCCAT
LoxP6ot 8	chr15:-99901206	CAGGTTGGCTCAACTCTTGAT	CTGTTGTCTGCCCTCCATTT
LoxP6ot 9	chr9:-34806732	GCCTCTACTCTGTCTTGCTCTA	TGCGGATGCAGTCTTGTTATAC
LoxP6ot 10	chr13:+103775807	ACCAACTGTAATGGGATCTGAC	CAGAGATAGGAGAACACACAGC
LoxP6ot 11	chr9:+89702030	CTTCATAGCTCCTCCCAATCAG	GAGAGTCAGGCAATACCAAAGA
LoxP6ot 12	chr17:+30398181	AATGCAAGCACTCCATCAAATC	TCTTGTCCTCAAGTGTCAAAGG

Table S7. Primer pairs to assess off target (ot) Cas9 activity during CRISPR-Cas9 insertion of loxP6.

Genomic coordinates for potential off target regions were listed in the NCBI37/mm9 build. These primer pairs were used in SURVEYOR mutation assays. Forward (F) and reverse (R) primers were abbreviated accordingly and cycling conditions using JumpStart Taq can be found in Table S2.
