# Supplementary material for: CRISPR/Cas9-Mediated Insertion of loxP Sites in the Mouse Dock7 Gene Provides an Effective Alternative to Use of Targeted Embryonic Stem Cells
Source: G3 (Bethesda). 2016 May 11;6(7):2051–61. doi: 10.1534/g3.116.030601 (PMC4938658; doi:10.1534/g3.116.030601)
Supplement: Supplemental Material [file supp_g3.116.030601_TableS8.pdf]

Target site	Injection type	Embryos transferred	Pups born/ birthrate	Non-transgenic	LoxP4	LoxP6	Both LoxP4 and LoxP6	Deletion between target sites	Null coat color
Dock7 cKO2	Cytoplasmic	174	20 (12%)	11/20 (55%)	7/20 (35%)	3/20 (15%)	1/20 (5%)	10/20 (50%)	5/19 (26%)

Table S8. Frequency of CRISPR-Cas9-mediated loxP insertion in the *Dock7* cKO2 model. The loxP sites described in *Dock7* cKO2 model were introduced by cytoplasmic injection. Mice were screened by genotyping, for loxP4 and loxP6 sites, deletion of DNA between the Cas9 cut sites (deletion of exon 3-7), and coat color. The number of mice with the indicated genotype or phenotype is listed compared to the total number of mice analyzed. The percentage of mice with the indicated genotype or phenotype is listed in parentheses. Null coat color indicates diluted coat color with the presence of a white belly spot. Non-transgenic mice are defined as mice without loxP insertion. Amplification of DNA from tail/toe clips was performed for loxP4 (loxP4A F/R or loxP4B F/R which provided identical results and could be used interchangeably), loxP6 (loxP6A F/R), and cKO2Δ (cKO2 Δ F/R) indicating deletion of exons 3-7.
