# Supplementary material for: CRISPR/Cas9-Mediated Insertion of loxP Sites in the Mouse Dock7 Gene Provides an Effective Alternative to Use of Targeted Embryonic Stem Cells
Source: G3 (Bethesda). 2016 May 11;6(7):2051–61. doi: 10.1534/g3.116.030601 (PMC4938658; doi:10.1534/g3.116.030601)
Supplement: Supplemental Material [file supp_g3.116.030601_TableS9.pdf]

Target site	Injection type	Non-transgenic (%)	5' LoxP (%)	3' LoxP (%)	5' and 3' LoxP (%)	Deletion (%)	Null coat color (%)	Correctly Targeted (%)
Dock7 cKO1-1	Pronuclear	2.7	1.3	2.7	1.3	2.7	0	0
Dock7 cKO1-2	Cytoplasmic	17.4	5.16	4.5	1.9	9.7	3.22	0.65
Dock7 cKO2	Cytoplasmic	6.3	4.0	1.72	0.57	5.7	2.9	0.57

Table S9. Frequency of CRISPR-Cas9-mediated loxP insertion in the *Dock7* locus. The data found in Table3 and Table S8 was calculated as a percentage based on the total number of transferred embryos.
