# Supplementary figures and images for: CRISPR/Cas9-Mediated Insertion of loxP Sites in the Mouse Dock7 Gene Provides an Effective Alternative to Use of Targeted Embryonic Stem Cells
Source: G3 (Bethesda). 2016 May 11;6(7):2051–61. doi: 10.1534/g3.116.030601 (PMC4938658; doi:10.1534/g3.116.030601)

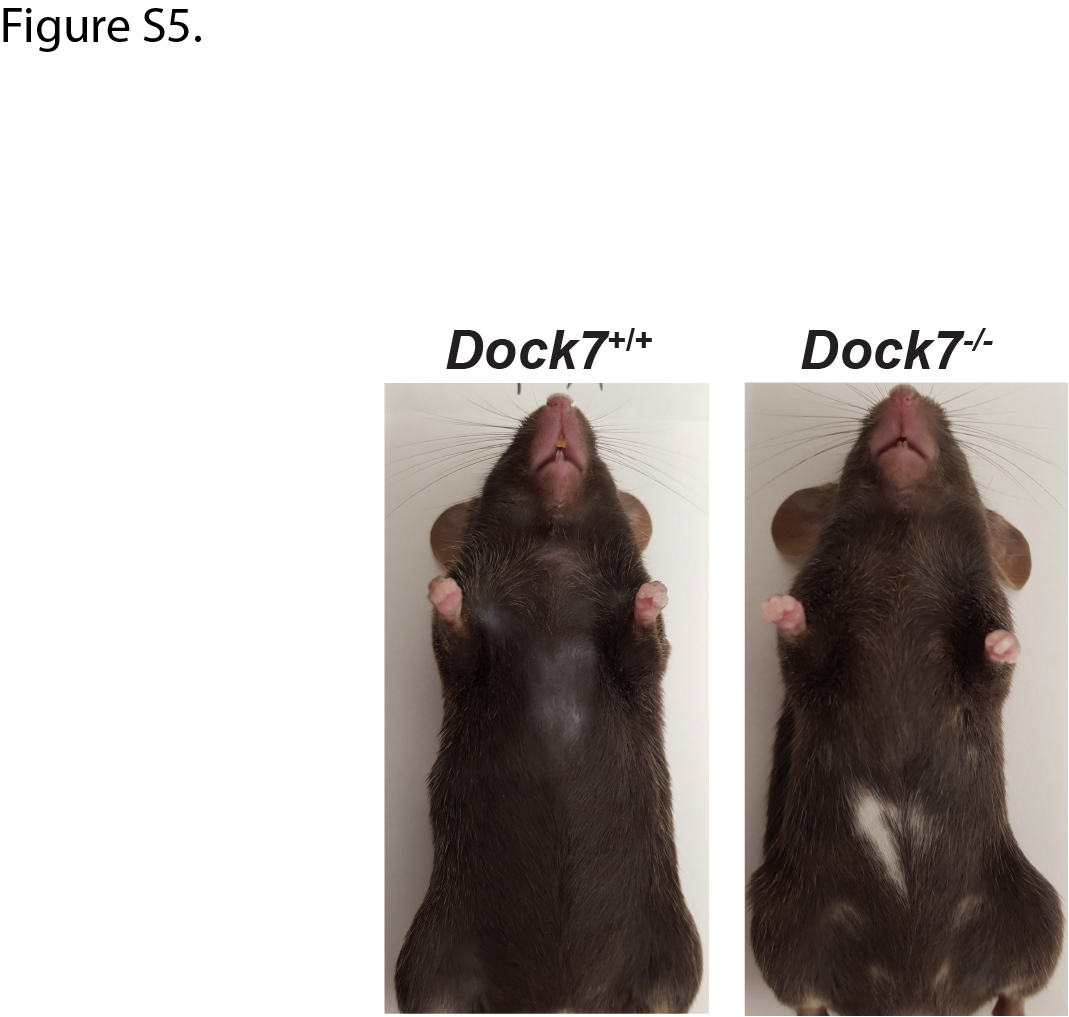

Supplement: Supplemental Material [file supp_g3.116.030601_FigureS5.tif]
